# Association of Healthy Diet with Recovery Time from COVID-19: Results from a Nationwide Cross-Sectional Study

**DOI:** 10.3390/ijerph18168248

**Published:** 2021-08-04

**Authors:** Faisal F. Alamri, Aslam Khan, Abdulaziz O. Alshehri, Ahmed Assiri, Shahd I. Khan, Leen A. Aldwihi, Munirah A. Alkathiri, Omar A. Almohammed, Ahmad M. Salamatullah, Amer S. Alali, Waleed Badoghaish, Abdulmajeed A. Alshamrani, Yazed AlRuthia, Faleh Alqahtani

**Affiliations:** 1 Basic Sciences Department, College of Science and Health Professions, King Saud bin Abdulaziz University for Health Sciences, Jeddah 22384, Saudi Arabia; alamrif@ksau-hs.edu.sa (F.F.A.); khanasl@ksau-hs.edu.sa (A.K.); 2 King Abdullah International Medical Research Center, Jeddah 22384, Saudi Arabia; 3 Department of Pharmacology and Toxicology, College of Pharmacy, King Saud University, Riyadh 11451, Saudi Arabia; 439105704@student.ksu.edu.sa; 4 General Directorate of Clinical Excellence, Ministry of Health, Riyadh 11176, Saudi Arabia; aasssiri@moh.gov.sa; 5 Department of Clinical Pharmacy, College of Pharmacy, King Saud University, Riyadh 11451, Saudi Arabia; 441204353@student.ksu.edu.sa (S.I.K.); 436202414@student.ksu.edu.sa (L.A.A.); 436200445@student.ksu.edu.sa (M.A.A.); yazeed@ksu.edu.sa (Y.A.); 6 Pharmaceutical Care Department, AlNoor Specialist Hospital, Ministry of Health, Makkah 24241, Saudi Arabia; 7 Pharmacoeconomics Research Unit, College of Pharmacy, King Saud University, Riyadh 11451, Saudi Arabia; 8 Department of Food Science and Nutrition, College of Food and Agricultural, King Saud University, Riyadh 11451, Saudi Arabia; asalamh@ksu.edu.sa; 9 Department of Pharmaceutics, College of Pharmacy, Prince Sattam bin Abdulaziz University, Al-Kharj 11942, Saudi Arabia; a.alali@psau.edu.sa; 10 Department of Internal Medicine, College of Medicine, University of Tabuk, Tabuk 71491, Saudi Arabia; wbadoghaish@ut.edu.sa; 11 Clinical Nutrition Department, Eradah Hospital and Mental Health, Ministry of Health, Alkharj 16259, Saudi Arabia; aalshamrani42@moh.gov.sa

**Keywords:** COVID-19, healthy diet, recovery time, dietary habits, hospitalization

## Abstract

The world is still in need of an effective therapy to treat coronavirus disease-19 (COVID-19). This cross-sectional study was conducted on COVID-19 survivors in Saudi Arabia to investigate the influence of a healthy diet on the recovery time from COVID-19. A questionnaire was developed to assess participants’ dietary habits, based on the 2015 Dutch food-based dietary guidelines. A total of 738 COVID-19 survivors participated in the study, of whom 237 (32.1%) were hospitalized for COVID-19 treatment while 501 (76.9%) were not hospitalized, and 320 (43.4%) were females and 418 (56.6%) were males. Overall, no significant difference was noted in healthy diet score between males and females; however, this score was significantly lower for Saudis compared to non-Saudis. Among the non-hospitalized patients, eating a more healthy diet was associated with a shorter duration of recovery (*p* < 0.05) and was significantly affected by gender (15.8 ± 9.3 male vs. 12.1 ± 8.9 female; *p* < 0.001) and marital status (12.1 ± 8.4 singles vs. 13.7 ± 9.3 married vs. 16.1 ± 11.8 divorced; *p* < 0.05). In contrast, no significant correlation was found with age or BMI. In this study, a more healthy diet was associated with a shorter duration of recovery from COVID-19. However, further studies are needed to thoroughly investigate the relationship between diet and recovery time from COVID-19.

## 1. Introduction

It is been over a year from the beginning of the coronavirus disease-19 (COVID-19) pandemic and a fourth wave has started in numerous countries [1,2]. The lack of an efficacious drug or sufficient supply of the newly developed vaccines for the severe acute respiratory syndrome coronavirus-2 (SARS-CoV-2) has further worsened the situation [3,4]. Many prospective drugs have been proposed, investigated, and used to treat COVID-19, such as hydroxychloroquine, chloroquine, ribavirin, remdesivir, favipiravir, and many others, but with limited or no success [5]. Accordingly, many vaccines were investigated in clinical trials all over the world [6]. Three vaccines for COVID-19 have been authorized for emergency use by the U.S. Food and Drug Administration [7]. In Saudi Arabia, the Saudi Food and Drug Authority has authorized four vaccines: the Pfizer–Biotech and Oxford/AstraZeneca, which are currently used by the Ministry of Health (MoH) in the national vaccination campaign, and the Moderna and Janssen COVID-19 vaccines, which are accepted for incoming travelers only [8]. Clinical trials suggested partial protection against COVID-19 after the first dose, and a second dose should be administered to develop enough immunity to fight COVID-19 [9].

Interestingly, the risk of developing COVID-19 varies between countries [10,11]. Beyond differences in incidence, noticeable differences in recovery rates among countries [4,10] have raised the question of whether COVID-19 is influenced by cultural factors such as nutrition and healthy dietary habits [12,13]. Nutrition-focused modifications could be used to minimize the impact of infectious diseases [14]. Healthy diet has been associated with strengthening immune responses and lowering the incidence of infectious diseases [15]. Moreover, healthy eating and balanced food choices support immunity and protect against unfavorable outcome [16]. A diet rich in saturated fats, sugars, and refined carbohydrates impair the innate and adaptive immunity, leading to chronic inflammation and weakened host defense against viruses. Thus, people who consume an unhealthy diet could be at an increased risk for severe COVID-19 and death [13,17]. Conversely, malnutrition is detrimental to health status, increasing the susceptibility to (and slowing the recovery from) infections [18,19]. Evidence has emphasized the vital role of dietary habits in altering patients’ prevalence and severity of, and the speed of recovery from, infectious diseases [14,20,21]. Comprehensive eating plans such as the traditional Mediterranean diet, which consist of healthy foods, can support the immune system’s response to infections [20]. Another diet plan that encourages similar healthy eating habits is offered by the 2015 Dutch food-based dietary guidelines [22], which recommend numerous food groups that are beneficial for preventing and treating infectious diseases [20,23,24,25,26,27,28,29]. The Dutch dietary guidelines describe what is currently known about the constituents of a healthy dietary pattern in order to prevent chronic diseases. The recommend dietary pattern involves eating more plant-based food and less animal-based food. For instance, it recommends eating at least 200 g of vegetables, 200 g of fruit, 15 g of unsalted nuts, 90 g of brown bread, whole-grain bread or other whole-grain products, and taking few portions of dairy produce daily while limiting the consumption of red meat, particularly processed meat, and minimizing the consumption of sugar-containing beverages [22]. Additionally, the Dutch guidelines have an important advantage over other measures, which is that they can be used fully or partially as a measurement tool to assess dietary patterns [30,31,32,33,34].

The COVID-19 pandemic has resulted in behavioral changes in food purchasing and consumption; people panic, and overstock food supplies as they fear food insecurity [35]. Some people started to have a healthy and balanced diet to maintain the correct nutrition status and reduce health risks. For instance, in Spain, people adopted healthier dietary habits and behaviors during the COVID-19 pandemic, reflected by a higher adherence to the Mediterranean diet [36]. On the other hand, the uncontrolled stress could lead some people toward comfort eating and overeating [37,38]. In the United Arab Emirates, increased food intake was the most commonly noticed unhealthy lifestyle change during the COVID-19 pandemic [39]. In Saudi Arabia, eating habits and the food industry have undergone many transitions in the last few decades because of economic growth and globalization [40]. Fast food consumption, less healthy dietary habits, and poor food choices in addition to physical inactivity resulted in increased obesity levels [41,42,43]. Moreover, during lockdown for the COVID-19 pandemic, unhealthier food intake habits and weight gain were noticed among Saudis [44,45]. 

Dietary patterns’ influence on recovery from infectious diseases in general has been investigated in previous studies but is not well documented [46]. Very few studies have been conducted to investigate the association between diet and recovery time from COVID-19, where it was shown that foods such as eggs, fish and seafood, meat, milk, vegetables and fruits, and nuts had a positive effect on COVID-19 recovery, especially in developed countries [47]. The purpose of this study was to explore the dietary habits for recovered COVID-19 patients in Saudi Arabia using the Dutch dietary guidelines. Then, the association between healthy diet scores and recovery time from COVID-19 was examined based on the participants’ demographic characteristics and hospitalization status: hospitalized vs. non-hospitalized for COVID-19 treatment.

## 2. Materials and Methods

### 2.1. Study Method and Subjects

A cross-sectional study was carried out from June to September 2020. Recently recovered COVID-19 patients were recruited from all five regions of Saudi Arabia: northern, southern, eastern, western, and central. Participants were randomly selected from the MoH database of patients who had COVID-19. Survivors were contacted by interviewers via phone call. After obtaining verbal consent for participation, patients’ data were collected and entered by interviewers into an electronic database. This study included adult (aged 18–80 years) participants who had recently recovered from COVID-19 that was confirmed by a polymerase chain reaction (PCR) test for SARS-CoV-2. Potential participants were excluded if they had non-symptomatic COVID-19, and in view of language barriers, the study excluded patients who were not able to understand Arabic, to avoid discrepancies during data collection.

### 2.2. Ethical Approval

This study was approved by the Ministry of Health (MoH) Central, Kingdom of Saudi Arabia (IRB No: 20-11E/17-06-2020). The need for written consent was waived by the ethical committee.

### 2.3. Development and Validation of Food Frequency Questionnaire

A food frequency questionnaire (FFQ) was constructed to reflect on the quantities recommended in the Dutch dietary guidelines [22]. The diet frequency measures used in the guidelines were adopted in the FFQ with some modifications to taken into consideration for Saudi dietary habits during the development and validation of the questionnaire [48]. Nine categories from the Dutch dietary guidelines were adopted to measure the overall dietary habits. A diet marked by healthy eating habits was assigned a score of “3” indicating full adherence, whereas a score of “0” indicated nonadherence and represented an unhealthy diet (Supplementary Table S1. Diet component questions and scores as per Dutch Guidelines). The FFQ investigated dietary habits relating to food intake, with the response options (“Daily”, “More than twice a week”, “Once a week”, “Once a month”, and “I don’t consume/eat it”) constructed to be consistent with the Dutch dietary guidelines and scored accordingly. The total scoring system ranged from 0 to 27, with higher scores representing a more healthy diet.

The face validity for the questionnaire was validated by expert healthcare professionals and biostatisticians, whereas the content validity was determined by a subject matter expert. The questionnaire was designed in Arabic and translated into English for validation by a bilingual expert not otherwise involved in the study. A pilot study was conducted that included 20 patients to confirm the questionnaire’s reliability, for validation, and to streamline the data collection process. Modifications were made to the questionnaire based on the results of the pilot study and feedback from the data collectors (interviewers) and patients. The privacy and confidentiality of patients’ information were maintained throughout the study, with soft and hard copies of the research data stored alike in a password-protected secure location accessible to the research team alone.

### 2.4. Statistical Analysis

The raw data obtained from the electronic database were transferred into an Excel spreadsheet for analysis. Missing values and outliers were identified and treated prior to the statistical analysis process. No missing values were found in the main variable of recovery period, and for the few cases of missing values, the imputation of an appropriate value of the mean was used to treat them prior to the analysis process. No principal analysis with imputed data was conducted. Since the sample size was large enough, Pearson’s correlation coefficient was calculated for the correlation between the recovery period and continuous variables.

For descriptive statistics, continuous variables were presented as the mean with standard deviation (SD), whereas categorical data were reported as frequency and percentages. Inferential statistics included *t*-testing for independent samples, one-way analysis of variance (ANOVA), and bivariate correlation analysis. All hypotheses were tested at the 5% level of significance. Descriptive and inferential statistical analysis was carried out using the Statistical Package for the Social Sciences (SPSS) version 25 (IBM Corp., Armonk, NY, USA).

## 3. Results

A total of 899 patients were contacted for participation in the study, of whom 768 consented to participate, for an overall response rate of 85.4%. Of the 768 respondents, data for 30 respondents were excluded from the analysis based on the inclusion/exclusion criteria.

### 3.1. Demographic Data

A total of 738 participants were included in the study. The majority (67.9%) were not admitted to hospitals. More than half of respondents were males (56.6%), Saudi citizens (75.7%), and married (67.8%). The mean (SD) age and BMI of respondents were 36.5 (±11.7) and 28.4 (±7.1), respectively. The proportions of hospitalization among non-Saudis and married participants were significantly higher. Similarly, the mean age and BMI of hospitalized patients were significantly higher (age 42.4 (±12.9) vs. 33.6 (±10.2); BMI 30.6 (±6.8) vs. 27.3 (±7.1)). Patient demographics are given in Table 1.

### 3.2. Diet Score and Recovery Time

The overall mean healthy diet score for all respondents was 15.9 (±3.7). The mean diet score for hospitalized patients was significantly higher (16.7 (±3.6) vs. 15.5 (±3.7); *p* < 0.01). The overall mean recovery time for all patients was 13.4 (±9.2). The mean recovery time for hospitalized patients was significantly longer (18.6 (±9.9) vs. 11.0 (±7.7); *p* < 0.01) (Table 2).

### 3.3. Comparison of Healthyl Diet Score with Basic Characteristics of Respondents

The results presented in Table 3 indicated no significant difference in the mean of healthy diet adherence scores between males and females. However, a significant difference was noted based on nationality and marital status, with the adherence to a healthy diet being significantly better in non-Saudi respondents (16.9 (±3.4) vs. 15.6 (±3.7); *p* < 0.01) and married individuals (16.3 (±3.3) vs. 15.6 (±3.7)).

### 3.4. The Relationship between Healthy Diet Score and Recovery Time from COVID-19

In non-hospitalized respondents, the results showed a negative correlation between recovery time and healthy diet score, indicating that recovery time decreased significantly with increases in healthy diet score. However, age and BMI were not correlated with the recovery time. On the other hand, diet score, age, and BMI were positively correlated with each other, which means that older respondents and those with a high BMI were more likely to adhere to a healthy diet. Conversely, a positive correlation was found between the recovery time and the healthy diet score among hospitalized participants, which means that those who adhered to a healthy diet were more likely to have a longer recovery time in comparison to their counterparts who do not. Moreover, older respondents and those with a high BMI were more likely to adhere to a healthy diet, just like their non-hospitalized counterparts. The correlation coefficients used to examine the associations between diet score, recovery time, age, and BMI for the two groups of respondents are shown in Table 4.

### 3.5. Comparison of Recovery Time with Basic Characteristics of Respondents

Overall, a statistically significant difference was noted in the recovery time based on gender and marital status. The mean recovery time for males was significantly shorter in the overall analysis for all patients, hospitalized patients, and non-hospitalized patients (overall 12.1 (±8.9) vs. 15.1 (±9.3); hospitalized: 16.9 (±9.8) vs. 20.7 (±9.7); non-hospitalized: 9.8 (±7.5) vs. 12.4 (±7.8); *p* < 0.001). The mean recovery time for non-hospitalized Saudi nationals was significantly longer (11.4 (±7.9) vs. 9.2 (±6.3), *p* < 0.05) but was significantly shorter than the mean in married and divorced/separated individuals (12.1 (±8.4) vs. 13.7 (±9.3) vs. 16.1 (±11.8), *p* < 0.05). Table 5 displays the association between recovery time and patient demographics for hospitalized and non-hospitalized participants.

## 4. Discussion

Currently, the world is facing a pandemic, as a new strain of the coronavirus has resulted in continuously increasing health challenges. The present study evaluated the impact of diet on the duration of recovery from COVID-19. The findings of the study indicate that the recovery duration for non-hospitalized patients was significantly shorter with increases in dietary score. Furthermore, the recovery period was noticeably shorter in male, younger, and unmarried patients.

Katona and Kotana-Apte have noted that “malnutrition is the primary cause of immunodeficiency worldwide” [14]. In a randomized controlled clinical trial, the effects of a healthy diet on the immunity of 1- to 4-year-old children (*n* = 118) against respiratory tract infection were investigated. Its outcomes suggested that adherence to a healthy nutritional regimen led to a shorter recovery duration, the reduced recurrence of infections, and the limited use of antibiotic courses. Additionally, studies of undernourished populations in Bangladesh and Angola reported increased prevalence of leprosy and malaria, respectively [46,49,50]. The body’s immunity against infectious diseases is dependent on nutrition, as divergence from a balanced dietary component alters the body’s capacity to fight off infection. The Dutch nutritional guidelines feature healthy diet components that positively influence immunity—including an appropriate proportion of micro- and macronutrients present in fruits [23,26,27], vegetables [24,25], seafood [28,29], and nuts [51], and that is essential for health when fighting infections [52].

In this study, the recovery period was significantly longer for hospitalized patients, even those who had better diet scores—perhaps reflecting the confounding factors encountered by the hospitalized population. For example, BMI and age were positively correlated with a healthy diet, suggesting that hospitalized patients with a high BMI (e.g., overweight and obese) and of an older age were more likely to adhere to a healthy diet. However, these very factors (e.g., high BMI and old age) are well-known factors to be associated with poor outcomes among COVID-19 patients. Therefore, the relationship between healthy diet and recovery time could be reversed if these factors were controlled [43]. Additionally, stress encountered in the hospital environment can be another reason behind hospitalized patients’ slower recovery, as stress and anxiety are known to alter the body’s immunity and increase its vulnerability to infection [53]. Moreover, hospitalized patients had a higher mean age and BMI than non-hospitalized patients, which may have put them at risk of slower recovery. The findings of the present study are in agreement with the previous literature indicating that older age [54] and obesity [55] prolong recovery time from infectious diseases, such as COVID-19. The immune system shifts throughout the lifetime, being optimal for young adults but gradually declining with age due to the dysregulation of immunity in response to increased oxidative stress [56]. Consequently, patients’ susceptibility to and severity of infections such as the common cold and influenza may increase with age [54]. Regardless of the healthy diet, the presence of obesity was associated with an approximately threefold increase in the risk of having severe COVID-19 and the association between obesity (or increasing BMI values) and greater COVID-19 severity remained significant even after adjusting for age, sex, smoking status, hypertension, diabetes, and dyslipidemia [57]. Furthermore, the relationship between a healthy diet and COVID-19 infection remains unclear so far, which necessitates further studies to examine this important association considering the lingering impact of the COVID-19 pandemic [58]

Moreover, the recovery period was notably shorter in males for both hospitalized and non-hospitalized patients, suggesting that gender differences might play a role in recovery from COVID-19. Other COVID-19 studies have concluded that males have a similar incidence rate and are at higher risk of severity and mortality than females [59,60]. However, gender differences in recovery time and response to COVID-19 have not been investigated. However, in line with the findings, a study by Johns Hopkins University has concluded that men might recover more quickly from influenza infections because of gender-based differences in the production of a key lung-healing protein [61]. Additionally, the recovery period from COVID-19 among Saudis was higher than non-Saudis. This could be attributed to the lower healthy diet score among Saudis, as observed in the results of our study. Moreover, this could be due to the previously reported unhealthy lifestyle, dietary behaviors, the higher cost of healthy foods, limited resources of information on healthy Arabic food choices, and limited physical activity among Saudis [42,62,63]. In addition, non-hospitalized patients had a lower diet score but recovered quicker than hospitalized patients. The faster recovery seen here is primarily attributed to the differences between the groups in age, BMI, and comorbidities (higher in the hospitalized patients’ group compared to non-hospitalized participants), as previous studies indicated [64,65].

Marital status also influenced the rate of recovery from COVID-19, with unmarried patients’ quicker recovery suggesting the impact of societal factors on response to infectious diseases. Studies have suggested that anxiety is common in the married population [66,67] as well as among divorced/widowed people [66,68]. Overall, the possibility that psychological health affects a person’s immunity against infectious diseases such as COVID-19 cannot be dismissed [69,70].

This study does have certain limitations. Variations in recovery time could exist because symptoms may present sooner or later after acquisition of the infection. As the population under study was uniformly from Saudi Arabia, the results of this study cannot be generalized to the rest of the world, where adherence to a healthy diet might be different. This limitation suggests the need for similar studies in other parts of the world. Socioeconomic status, represented by health literacy, educational achievement, and income level, might have varied among participants more than realized and was not controlled for in this study. Future research should do so, however, because this factor may affect participants’ malnutrition status. In retrospective studies, it is very difficult to track the diet consumption precisely. Food composition tables are the best way to assess healthy diet consumption, but these are not available from authorities in Saudi Arabia. Therefore, the FFQ was the best alternative in this case; it is a simple, valid, and reproducible tool to study dietary intake in epidemiological studies [71,72]. Moreover, when building the FFQ, we adopted items from the Dutch dietary guidelines for multiple reasons. First, when compared to other recommendations and guidelines such as the Australian, American, and Nordic dietary guidelines, the Dutch guideline has the advantage of data acquisition feasibility, for both investigators and participants. For investigators, descriptive information is converted to values ranking patients dining habits based on its healthiness, as the scoring system converts the type and amount of diet into values and participants with higher values represent better adherence to healthy diet habits. For participants, the Dutch guideline is simple and straightforward; as it is exclusively based on food consumption, whereas other guidelines are more complicated, as they incorporate nutrients consumption. Hence, the Dutch guideline is expected to have a lower chance of errors and would facilitate the process of data collection in large populations. Additionally, the Dutch dietary guidelines were used to define healthy diet habits, which was defined by the sufficient intake of different foods from nine food categories. All or some of the categories in the Dutch dietary guidelines can be adopted to assess healthy diet habits among participants [30,31,32,33,34]; in our study, we used the majority of categories in the guidelines. Besides that, the Healthy Food Palm for Saudi people indicates proportions/quantities of daily intake, but previous studies show that most Saudis do not adhere to the Healthy Food Palm recommended ranges [73]. It was assumed that participants would not know the actual amount of salt and meat they consume on daily bases but fast foods and ready to eat foods are rich sources of salt [22,74]. Thus, participants’ daily consumption of salt or meat were not estimated through direct questions; it was rather projected through asking participants about the amount of fast food consumed for the salt and the frequency of red meat meals on weekly bases and these were used as a proxy for the healthy amount of salt and red meat consumed on daily bases.

Lastly, it was not clear from our data why people with a higher BMI had a healthier diet score. The FFQ has its limitations as the information in these questionnaires are mainly based on participants’ recall of their usual diet and because of the predefined food list, some information about the foods actually eaten may be missed or overlooked [75]. Moreover, most FFQs overestimate nutrient intake and food consumption compared to other methods [76,77,78,79]. Therefore, we recommend future research to examine the validity of the FFQs against other dietary assessment methods. This will help deepen knowledge on the possible effect of participants’ characteristics on reporting food consumption which may explain differences among participants in food consumption, including BMI. Additionally, this could be a limitation in the measurement of BMI, since it has the potential for inaccuracy, particularly when it is based on self-reported height and weight, as in our case. Provided that BMI does not measure adipose tissue, people with significant lean body mass might be classified as overweight or obese while they would likely have a low body fat percentage [80,81].

## 5. Conclusions

In conclusion, the present study suggests that a healthy and balanced nutritional regimen could be of help in fighting off COVID-19 in non-hospitalized patients. Patients’ recovery rate was inversely related to their diet score while accounting for the mentioned societal and psychological factors. Even so, additional investigation is needed of a comparatively larger population from other countries to further validate the outcomes of this study.

## Figures and Tables

**Table 1:** Patient demographics ^†^.

Variables	Overall, Sample N = 738	Non-Hospitalized N = 501	Hospitalized N = 237	*p*-Value *
Age, mean (SD)	36.5 (11.9)	33.6 (10.2)	42.4 (12.9)	<0.0001
BMI, mean (SD)	28.4 (7.1)	27.3 (7.1)	30.6 (6.8)	<0.0001
Gender	-	-	-	-
Male	418 (56.6)	286 (57.1)	132 (55.7)	0.7542
Female	320 (43.4)	215 (42.9)	105 (44.3)	-
Nationality	-	-	-	-
Saudi	559 (75.7)	409 (81.6)	150 (63.3)	0.0012
Non-Saudi	179 (24.3)	92 (18.4)	87 (36.7)	-
Marital status	-	-	-	-
Single	199 (27.0)	168 (33.5)	31 (13.1)	0.0015
Married	500 (67.8)	312 (62.3)	188 (79.3)	-
Divorced	26 (3.5)	18 (3.6)	8 (3.4)	-
Widow/widower	13 (1.7)	3 (0.6)	10 (4.2)	-

^†^ Data in this table were previously published by the team in another research including the same participants in this research [21]. Data presented as frequency (%) unless otherwise indicated. * *p*-values were from *t*-test for continuous data (age and BMI) and chi-squared test for categorical data.

**Table 2:** Means and standard deviation (SD) for healthy diet score and recovery time (days).

Variables	All Patients	Non-Hospitalized	Hospitalized	*p*-Value *
Healthy Diet (score)	15.9 (3.7)	15.5 (3.7)	16.7 (3.6)	<0.001
Recovery time (days)	13.4 (9.2)	11.0 (7.7)	18.6 (9.9)	<0.001

Data presented as means with standard deviation (SD). * *p*-values were from *t*-test.

**Table 3:** Healthy diet score by basic demographical characteristics of respondents.

Variables	All Patients		Non-Hospitalized		Hospitalized	
	Mean (SD)	*p*-Value	Mean (SD)	*p*-Value	Mean (SD)	*p*-Value
**Gender**	-	0.447	-	0.075	-	0.169
Male	15.8 (3.7)	-	15.3 (3.7)	-	17.0 (3.3)	-
Female	16.0 (3.7)	-	15.9 (3.6)	-	16.4 (3.6)	-
**Nationality**	-	0.001 **	-	0.001 **	-	0.079
Saudi	15.6 (3.7)	-	15.3 (3.7)	-	16.4 (3.6)	-
Non-Saudi	16.9 (3.4)	-	16.6 (3.3)	-	17.2 (3.4)	-
**Marital status**	-	0.001 **	-	0.001 **	-	0.012 *
Single	15.6 (3.7)	-	14.4 (3.4)	-	15.1 (4.2)	-
Married	16.9 (3.3)	-	16.1 (3.6)	-	17.1 (3.4)	-
Divorced	15.5 (4.7)	-	15.9 (5.3)	-	14.8 (3.7)	-
Widow/widower	16.4 (2.5)	-	16.0 (0.1)	-	16.5 (2.9)	-

Data presented as means with standard deviation (SD) and *p*-values were from *t*- or ANOVA test. * Represents *p*-value significant at < 0.05 and ** represents *p*-value significant at < 0.01.

**Table 4:** Correlation matrix of healthy diet score, recovery time (days), age, and BMI for hospitalized and non-hospitalized respondents.

	Non-Hospitalized				Hospitalized			
	Recovery Time	Diet Score	Age	BMI	Recovery Time	Diet Score	Age	BMI
Recovery time	1				1			
Diet score	−0.088 *	1			0.161 *	1		
Age	−0.026	0.335 **	1		0.127 *	0.347 **	1	
BMI	0.088	0.186 **	0.258 **	1	0.102	0.006	0.263 **	1

* Represents *p*-value significant at < 0.05 and ** represents *p*-value significant at < 0.01.

**Table 5:** Recovery time (days) by basic demographical characteristics of respondents.

Variables	All Patients		Non-Hospitalized		Hospitalized	
	Mean (SD)	*p*-Value *	Mean (SD)	*p*-Value	Mean (SD)	*p*-Value
**Gender**	-	0.001 **	-	0.001**	-	0.004 **
Male	12.1 (8.9)	-	9.8 (7.5)	-	16.9 (9.8)	-
Female	15.1 (9.3)	-	12.4 (7.8)	-	20.7 (9.7)	-
**Nationality**	-	0.420	-	0.017 *	-	0.781
Saudi	13.3 (9.0)	-	11.4 (7.9)	-	18.5 (9.8)	-
Non-Saudi	13.9 (9.6)	-	9.2 (6.3)	-	18.8 (10.1)	-
**Marital status**	-	0.041 *	-	0.397	-	0.871
Single	12.1 (8.4)	-	10.8 (7.1)	-	18.9 (11.4)	-
Married	13.7 (9.3)	-	10.9 (7.8)	-	18.5 (9.7)	-
Divorced	16.1 (11.8)	-	13.9 (11.6)	-	20.9 (11.9)	-
Widow/widower	16.0 (8.3)	-	12.7 (2.3)	-	17.0 (9.4)	-

Data presented as means with standard deviation (SD) and *p*-values were from *t*- or ANOVA test. * Represents *p*-value significant at <0.05 and ** represents *p*-value significant at <0.01.

## Data Availability

The data are available upon reasonable request from the corresponding author.

## Supplementary Materials

The following are available online at https://www.mdpi.com/article/10.3390/ijerph18168248/s1, Table S1: Diet component questions and scores as per Dutch Guidelines
