# Supplementary material for: Association of Healthy Diet with Recovery Time from COVID-19: Results from a Nationwide Cross-Sectional Study
Source: Int J Environ Res Public Health. 2021 Aug 4;18(16):8248. doi: 10.3390/ijerph18168248 (PMC8394364; doi:10.3390/ijerph18168248)
Supplement: Supplementary file 1 [file ijerph-18-08248-s001.zip › ijerph-1283742-supplementary.pdf]

Supplemantary Table S1. Diet component questions and scores as per Dutch Guidelines

Diet Components	Questions related to our component	Conversion and comments	Ordinal Scale for our Food Frequency Questionnaire
1. Fruits	I eat fruits such as watermelon, orange, or bananas	Once a day consider as (≥100g/day) and more than once a day consider as (≥200 g/d)	More than once a day = 3<br>Once a day =2<br>Twice a week/More than twice a week = 1<br>I don't eat it = 0
2. Vegetables	I eat vegetables such as cucumbers, tomatoes, and carrots	Once a day consider as (≥100g/day) and more than once a day consider as (≥200 g/d)	More than once a day = 3<br>Once a day =2<br>Twice a week/More than twice a week = 1<br>I don't eat it = 0
3. Eat at least 90 g brown bread, whole meal bread or other wholegrain products daily.	Which source of carbohydrate do you eat?	once a day consider as (≥90g/day)	Brown Flour or Oats = 3<br>White Flour=0
4. Eat at least 15 g of unsalted nuts daily	How many times you eat nuts as a healthy source of fat (nuts, almonds, pistachios, hazelnuts, peanuts, and sunflower seed)?	Once a day consider as (≥15g/day)	Daily = 3<br>More than twice a week/Once a week=2<br>Once a month=1<br>I don't eat it=0
5. Eat one serving of fish, preferably oily fish, weekly	How many times you eat seafood?	once a week consider as covering DUTCH Guideline, more than once is better as they have mention in guideline	Daily/More than twice/once a week = 3<br>Twice a month =2<br>Once a month or I don't eat it = 0
6. Replace butter, hard margarines and cooking fats by soft margarines, liquid cooking fats and vegetable oils	Which sources of oil do you use permanently to eat?	Olive Oil<br>Canola Oil<br>Corn Oil (Healthy choice)	Olive Oil/Other plant sources Oil = 3<br>Animals' source Oil = 0
7. Limit the consumption of red meat	How often do you eat red meat (Sheep, Calf, Camel etc.)?	Only these options score as 3<br>Once a month<br>I don't eat it	Daily =0<br>More than twice a week = 1<br>Once a week =2<br>Once a month/I don't eat it = 3

Diet Components	Questions related to our component	Conversion and comments	Ordinal Scale for our Food Frequency Questionnaire
8. Limit salt intake to 6 g daily	How many times do you eat fast food?	(purchased food) considered as source of salt as they mention in DUTCH guideline	Daily =0<br>More than twice a week = 1<br>Once a week =2<br>Once a month/I don't eat it = 3
9. Take a few portions of dairy produce daily, including milk or yogurt	If you're using dairy products, what is the quantity?	Only these options score as 3<br>2 cups a day<br>More than 2 cups a day	2 cups a day/More than 2 cups a day =3<br>1 cup a day=2<br>One cup a week=1<br>I don't eat it= 0

A comprehensive healthy diet score was obtained by adding the scores that were assigned to the daily intake of the above mentioned 9 diary components. The healthy diet score ranges from 0 (no adherence) to 27 (Full adherence) points, as per the Dutch dietary guideline.
The red highlighted comments were excluded in the study.
Micronutrients supplements like vitamins D, B12 and C were analyzed separately.

Dutch Dietary Guidelines Recommendation.

Higher consumption recommended

● Eat at least 200 g of *vegetables* and at least 200 g of *fruit* daily.
● Eat at least 90 g brown bread, whole meal bread or other wholegrain products daily.
● Eat legumes weekly.
● Eat at least 15 g of unsalted nuts daily.
● Eat one serving of fish, preferably oily fish, weekly.
● Drink three cups of tea daily.

Replacement recommended

● Replace refined cereal products by whole-grain products.
● Replace butter, hard margarines and cooking fats by soft margarines, liquid cooking fats and vegetable oils.
● Replace unfiltered coffee by filtered coffee.

Limitation recommended

● Limit the consumption of red meat, particularly processed meat.
● Minimize the consumption of sugar-containing beverages.
● Do not drink alcohol or no more than one glass daily.
● Limit salt intake to 6 g daily.
● Nutrient supplements are not needed, except for people who belong to a group for which supplementation applies.

Maintenance of current consumption recommended

● Take a few portions of dairy produce daily, including milk or yogurt.
